# Interdisciplinary perspectives on multi-risk anticipatory action

**DOI:** 10.1016/j.isci.2026.115618

**Published:** 2026-04-08

**Authors:** Tesse A. de Boer, Alessia Matanó, Evan Easton-Calabria, Tilly Alcayna, Mirianna Budimir, Marleen C. de Ruiter, Madeline Ewbank, Muhammad Fawwad, Debora Gonzalez, Catalina Jaime, Lindsey Jones, Kim Kristensen, Brenda Lazarus, Rodrigo Mena, Edward Parkinson, Robert Šakić Trogrlić, Beth Simons, Timothy Tiggeloven, Marc J.C. van den Homberg, Cees J. van Westen, Martha M. Vogel, Andy Wheatley, Christopher J. White, Andrew Kruczkiewicz

**Affiliations:** 1 Red Cross Red Crescent Climate Centre, 2593 HT The Hague, the Netherlands; 2 Institute for Environmental Studies, Vrije Universiteit Amsterdam, 1081 HV Amsterdam, the Netherlands; 3 Tufts University, Medford, MA 02155, USA; 4 London School of Hygiene and Tropical Medicine, London WC1E 7HT, UK; 5 Global Health Resilience Group, Barcelona Supercomputing Center, Barcelona 08034, Spain; 6 Practical Action, Rugby CV21 2SD, UK; 7 Welthungerhilfe Anticipatory Humanitarian Action Facility (WAHAFA), Welthungerhilfe, Bonn 53173, Germany; 8 Aktion gegen den Hunger (ACF), Berlin 53173, Germany; 9 Faculty of Geo-Information Science and Earth Observation (ITC), University of Twente, Enschede 7522 NH, the Netherlands; 10 The Risk-informed Early Action Partnership (REAP) Secretariat hosted at the International Federation of Red Cross and Red Crescent Societies, Geneva 1209, Switzerland; 11 London School of Economics and Political Science, London WC2A 2AE, UK; 12 Food and Agriculture Organization of the United Nations, Subregional Office for Eastern Africa, United Nations Office at Nairobi, Nairobi 00200, Kenya; 13 The Hague Humanitarian Studies Centre, Institute of Social Studies (ISS) of Erasmus University Rotterdam, The Hague 2502 LT, the Netherlands; 14 Start Network, London EC1V 9DD, UK; 15 Systemic Risk and Resilience, International Institute for Applied Systems Analysis, Laxenburg 2361, Austria; 16 CMCC Foundation - Euro-Mediterranean Center on Climate Change, Venice 30121, Italy; 17 The Netherlands Red Cross’ Data and Digital Team 510, The Hague 2593 HT, the Netherlands; 18 International Committee of the Red Cross (ICRC), Geneva 1202, Switzerland; 19 Department of Civil and Environmental Engineering, University of Strathclyde, Glasgow G1 1XQ, UK; 20 National Center for Disaster Preparedness, Columbia Climate School, Columbia University, New York, NY 10025, USA

**Keywords:** hazard communication, social sciences, interdisciplinary application studies

## Abstract

Anticipatory action (AA) has transformed how humanitarian actors respond to forecasted crises, yet most systems remain built around single hazards. This perspective argues that to stay effective in a world where climate, conflict, and economic shocks increasingly intersect, AA must evolve toward a multi-risk approach. Drawing on a review of 105 active frameworks in 2023 and 154 in 2024 from the Anticipation Hub’s 2024 and 2025 global overview reports, expert consultations, and 17 interviews, we examine how practitioners and scientists are beginning to bridge this gap. Emerging innovations—such as multi-risk analysis informing AA design, scenario-based triggers, conflict-sensitive planning, and adaptive financing—offer promising pathways, but current systems still struggle to capture dynamic vulnerabilities and interactions between risks. Advancing AA will require embracing uncertainty and redesigning systems to learn, adapt, and act across interconnected risks—moving from anticipating single hazards to anticipating intersected crises.

## Graphical abstract

**Figure:**
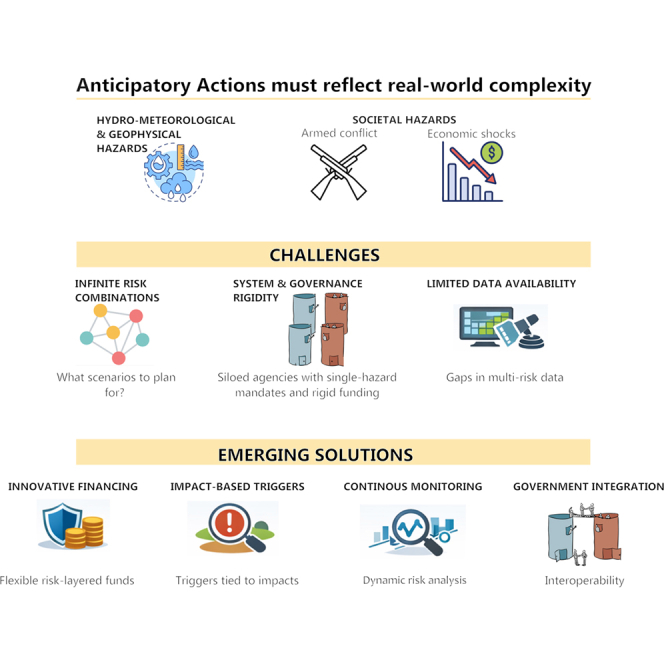

## Introduction: Why multi-risk matters for anticipatory action

Multi-risk (MR) events have become more frequent in recent years, driven not only by hydro-meteorological hazards like droughts, extreme winds, and floods,^1^^,^^2^ but also by intensifying armed conflict, violence, and weak governance.^3^^,^^4^ These societal hazards^5^ and vulnerability factors increasingly intersect with hydro-meteorological hazards, amplifying their impacts, particularly in regions already grappling with humanitarian crises.^6^^,^^7^^,^^8^ Between 2000 and 2018, events involving multiple hydro-meteorological hazards accounted for 78% of total damages, 83% of affected populations, and 69% of total deaths in reported disasters.^9^ Yet these figures often mask the complex interplay among hazards, ongoing conflict, displacement, and state fragility. Looking ahead, these MRs are projected to intensify under the current climate and geopolitical trajectories.^10^^,^^11^^,^^12^

Anticipatory action (AA) is one approach available to disaster risk management practitioners to mitigate disaster impacts by acting before they occur. It is defined as “acting ahead of a predicted hazardous event to prevent or reduce impacts on lives and livelihoods and humanitarian needs before they fully unfold”.^13^ AA is the most effective when activities, triggers, and decision-making rules are pre-agreed, guaranteeing fast release of pre-arranged funding. Despite the increasingly complex operational reality, AA frameworks, which define the triggers, pre-agreed actions, and roles for specific events, still primarily address risks individually.^14^^,^^15^ While AA has demonstrated value in mitigating predictable impacts, anecdotal evidence^15^^,^^16^^,^^17^^,^^18^^,^^19^^,^^20^ suggests that the current single-hazard orientation is insufficient in MR contexts, limiting its effectiveness and, in some cases, exposing communities to harm by neglecting interacting risks. While there is a broad call for more MR approaches in AA,^21^ AA frameworks that explicitly adopt a MR perspective are in the minority, and there is a lack of clarity in the AA community on what should be done.

This perspective assesses the current capacity of AA to address MR events and suggests a way forward for integrating MR approaches in AA. We argue that integrating MR approaches into AA is essential to capture overlapping, cascading, and nonlinear impacts, with important implications for framework design. Flexible financing and stronger links between AA, preparedness, and mitigation are also critical, given that communities and disaster risk management professionals may simultaneously find themselves in multiple phases of the disaster risk management continuum during consecutive or compounding events. Bridging science and practice, by aligning advances in MR analysis with practitioner insights, will be central to building AA that is adaptive, context-specific, and better suited to the realities of MR crises. This does not mean each AA framework must cover every possible risk, but that MR analysis, flexible contingency planning, and dynamic vulnerability information are needed to anticipate and mitigate peaks in impacts and operational challenges. We use the term “multi-risk” following Zschau^22^ to emphasize not only hazards but also the critical roles of vulnerability and exposure in disaster risk management and AA, focusing on the interactions most relevant from the perspective of AA practitioners.

This perspective reflects the views of the Anticipation Hub’s Multi-Risk Working Group and is informed by a small qualitative study combining desk analysis of 107 AA frameworks activated in 2023 and 154 in 2024, expert elicitation, and 17 interviews with key informants on implementation, financing, and research (see supplemental information for details). In this paper, the term “expert elicitation” is used in a qualitative sense to describe structured engagement with experts through interviews and facilitated discussions, rather than formal elicitation methods. While incorporating diverse geographic perspectives, the study is not exhaustive. The desk-based review of current practices drew on AA frameworks featured in the Anticipation Hub’s annual overview reports (2024 and 2025)^21^^,^^23^ and was conducted iteratively alongside expert elicitations and interviews to ensure cross-validation and integration of insights. In the following sections, we use these diverse sources to discuss current approaches to identifying and integrating risks in AA, challenges and opportunities for advancing MR approaches, and implications and recommendations for practitioners, researchers, and donor organizations. The conclusions and recommendations also speak more broadly to disaster risk management science, policy, and financing, offering pathways to more adaptive, evidence-based, and context-specific interventions.

## Current practice: How MR is currently operationalized in AA

MR approaches are increasingly relevant for AA, but implementation remains the exception rather than the norm. Broadly defined, MR integration in disaster risk management means recognition of the interaction of multiple risks and their complexity, enabling a more comprehensive risk understanding,^24^ and thus, is highly relevant for many contexts in which AA is implemented. Emerging practices demonstrate this potential—for example, AA frameworks addressing food insecurity,^25^ epidemics,^26^ or combined events such as *dzud* in Mongolia^27^—but they remain isolated cases. Even when existing single-hazard frameworks incorporate MR dynamics, for example, through scenario-testing of triggers and operational procedures, this is rarely done systematically. Currently, there is no institution that has standardized guidance on integrating MR into AA frameworks and their implementation.^15^^,^^16^^,^^20^^,^^28^

**Figure 1:**
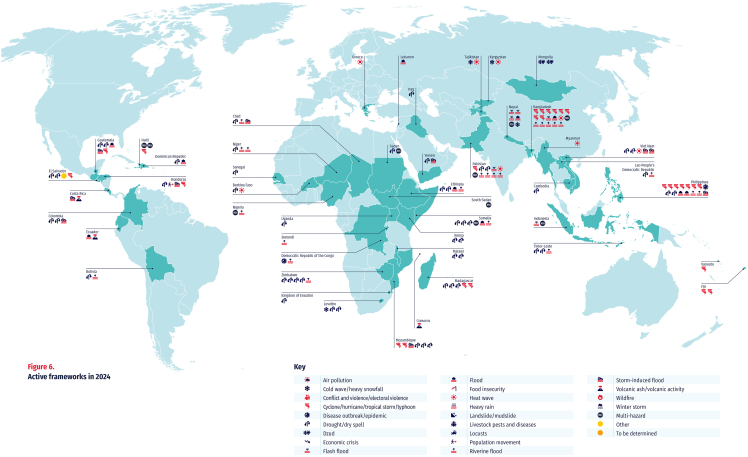
Active frameworks in 2024 as per the Anticipation Hub Global Overview Credit: Anticipation Hub.^23^

Figure 1 provides an overview of active frameworks in 2024. Literature exploring global developments of multi-hazard early warning systems (MHEWSs) also emphasizes that current trends indicate more development of separate forecasting systems in parallel (referred to as “multiple hazard” instead of “multi-hazard” systems, for example), while advancement toward interconnected forecasting systems is not taking place to the extent that it arguably should.^29^

Our analysis of active AA frameworks and AA experiences without pre-agreed plans in 2023 and 2024 confirms that MR integration in AA remains limited and uneven, with progress toward more impact-based AA and more “multiple hazard” frameworks. This is particularly evident through a subset of examples of frameworks and AA activations that capture interactions of compound and cascading risks. Despite rapid growth of AA over the last decade, most frameworks are also still designed for climate-related extremes, failing to prepare for scenarios where multiple different hazard types^5^ (such as hydro-meteorological, biological, and societal hazards) converge. We analyzed the Anticipation Hub’s global overviews of active AA frameworks in 2023 and 2024,^21^^,^^23^ and combined this with information from expert elicitation to understand how MR is currently integrated in AA. As part of this, we sought to recognize the diversity of risks, organizations, and funding mechanisms in the sector. The data from this analysis revealed three main categories of MR integration, each with distinct characteristics, challenges and innovations: (1) single-hazard frameworks (analyzing risks, selecting early warning sources, and determining specific triggers and actions for one hazard, such as a flood), (2) AA without pre-agreed frameworks, and, (3) frameworks for MR events. Within the last category, two subtypes emerged: (3A) frameworks for impacts and/or cascading and compounding risks, and (3B) frameworks that coordinate multiple single-hazard plans without cross-hazard integration. The key differences lie in what is being anticipated—hazards or their impacts—and in how frameworks are structured, governed, and formalized, which in turn shapes how MR dynamics are addressed. This overview is summarized in Table 1.

**Table 1:** Synthesis of insights into the types of AA currently implemented and how MR is integrated

Framework type and prevalence	How MR is addressed	Challenges/limitations	Emerging innovations and good practices examples
Type 1: single-hazard frameworks 2023: 92 (86%) 2024: 124 (81%)	Primarily address one hazard, with limited reference to cascading or secondary impacts	Separate frameworks often lack operational linkages and cannot effectively address co-occurring hazards	Incremental inclusion of secondary impacts (e.g., disease after floods or drought) and linked triggers; some efforts to connect stand-alone frameworks through scenario planning and adapted operational procedures (e.g., Madagascar Red Cross^30^).
Type 2: AA without pre-agreed frameworks (flexible funding and crisis modifiers) Not included in framework totals	Early actions taken without pre-set triggers, relying on contextual risk assessments and flexible funding; often supported by pooled funds (e.g., Start Fund) or internal crisis modifiers	Ad hoc implementation, weak prepositioning, and preparedness limit application for large-scale events, uncertainty in funding allocation, and procedures and limited scale	Use of flexible financing instruments for MR early action; AA alerts within the Start Fund are increasingly applied to MR events, linking climatic and societal risks (e.g., conflict or economic shocks), as seen in activations in Angola and Costa Rica;^31^^,^^32^^,^^33^ in parallel, crisis modifiers are being used to embed anticipatory elements within ongoing humanitarian programmes in conflict settings (e.g., ICRC Somalia), enabling early action for compounded impacts in the absence of a dedicated AA fund^34^
Type 3A:.focus on cascading or compounding risks 2023: 12 (11%) 2024: 23 (15%)	Frameworks link hazards in causal chains (e.g., cyclone → flood → disease) or anticipate MR impacts such as food insecurity, displacement, or economic losses	Complex monitoring and coordination across sectors; data-intensive and often limited to pilot contexts or agency-specific mandates	Growing use of compound-risk modeling in frameworks and some exploration of dynamic thresholds, albeit not included in frameworks yet; new frameworks for crises with multiple risk drivers, such as epidemics,^26^^,^^35^^,^^36^ food insecurity^25^ and malnutrition,^37^ and displacement^38^
Type 3B: frameworks coordinating multiple single-hazard plans without cross-hazard integration. 2023: 3 (3%) 2024: 7 (4%)	Multiple single-hazard AA plans embedded within a single overarching framework, characterized by shared governance arrangements (e.g., a national disaster management authority or inter-agency coordination body) that oversees decision-making, prioritization, and activation across hazards; while governance and coordination are centralized, hazard analysis, triggers and actions remain hazard-specific and are not causally or analytically integrated	Fragmented funding and coordination in the case of inter-agency plans; limited coherence, or scalability; does not inherently capture MR interactions and cascading dynamics, resulting in similar challenges as type 1—frameworks typically do not have shared or linked triggers between hazards	Some convergence under shared national risk platforms and regional roadmaps; efforts to harmonize funding triggers and inter-agency plans in, for example, Bangladesh and Ethiopia.^39^ WFP and Save the Children multi-hazard plans such as the Pakistan multi-hazard framework for drought, floods, and heatwave^23^—although further steps could be taken to connect plans to cover compounding and cascading impacts

This shows the diversity in approaches and aims to capture practices across the sector. The information is based on a desk-based review of 107 frameworks activated in 2023 and 154 in 2024, reported in the 2024 and 2025 global overview reports of the Anticipation Hub^21^^,^^23^, and subsequent interviews and expert elicitation.

Across all AA frameworks reviewed for 2023–2024, single-hazard systems (type 1) remain dominant. However, the number of MR frameworks (type 3) nearly doubled, with most focusing on cascading or impact-driven risks—for example, Honduras’s hurricane-induced flood framework^40^ and Mongolia’s *dzud* plans linking drought and cold extremes.^27^^,^^41^ This type of AA is especially expanding in Latin America, East Africa, and Southeast Asia. A growing subset under type 3A also integrates impact-based triggers tied to food insecurity or displacement, such as Action Against Hunger’s work on malnutrition^37^ and various AA frameworks for displacement by Danish Refugee Council and the Red Cross. Meanwhile, flexible mechanisms (type 2), such as the Start Fund and crisis modifiers, continue to support AA and early response in compounding crises but remain ad hoc. Type 3B represents a governance-level integration rather than a risk-analytic integration, offering coordination and efficiency gains without requiring joint hazard modeling or compound triggers. Overall, there are more dynamic, impact-oriented approaches (focusing on anticipating and preventing impacts such as epidemics and food insecurity) and greater integration into the governance of single-hazard frameworks.

## Cross-cutting challenges in addressing MR across AA

Despite advancements in MR integration, cascading and compounding events continue to challenge all phases of AA—from risk analysis and trigger design to early action planning and financing (as also highlighted by expert consultations within the Anticipation Hub and key informant interviews). Recent activations illustrate this fragility. In Mozambique (2021), actions planned for cyclone Eloise were derailed by the cumulative effects of Chalane, Idai (2019) and COVID-19.^17^^,^^42^ In Ethiopia, the impact of drought AA was found to be undermined by lingering impacts from previous shocks and conflict.^16^ In the Philippines, market-based cash interventions, implemented as early actions for cyclones, became unviable due to market disruption and inflation driven by preceding events.^28^ More broadly, a review of recent AA activations across the Red Cross and the Red Crescent Movement identified compound risks as a major implementation challenge, especially where evolving risk conditions substantially altered the operational context between framework design and implementation.^20^ These examples highlight the practical consequences of MRs and underscore the importance of underlying challenges in risk analysis, trigger design, early actions, and financing.

Risk analyses and triggers largely remain narrowly focused on single hydro-meteorological hazards, with societal hazards and vulnerability factors treated mainly as context rather than as integral drivers of activation. Advances in operational multi-hazard early warning and impact-based forecasting systems are promising, yet still fail to capture the interactions between risks, vulnerabilities, and exposures that drive disaster impacts.^43^^,^^44^ This narrow focus is partly due to limited monitoring data and the strategic choice to keep systems operationally simple. Indeed, while experts acknowledged that most humanitarian settings are MR by nature, balancing analytical complexity with practical usability remains difficult.

Designing and monitoring triggers for multiple interconnected hazards is especially challenging, and often infeasible where forecasting capacity and data access are weak. This challenge is heightened in countries with high income disparity and poverty levels, where forecasting capacity is often low.^45^ However, promising examples are beginning to emerge, including the integrated inter-agency cyclone and cascading floods AA framework in Bangladesh, as well as pilot initiatives across organizations addressing AA for biological hazards with explicit attention to compounding and cascading risks and MR impacts such as displacement.^26^^,^^38^^,^^46^

Concerning the selection of early actions, current AA frameworks (current practice: how MR is currently operationalized in AA) often account for cascading risks (e.g., flood followed by landslides). However, co-occurring or consecutive events are rarely covered, and practitioners struggle to anticipate how hazards might overlap or evolve beyond their technical mandates. For example, experts flagged the risk of unintended changes in risks through AA—reinforcing shelters for cyclones can increase exposure to heat stress depending on the material used, evacuation can increase the risk of infectious diseases in shelters (e.g., COVID-19), and cash support for one hazard may increase the risk of robbery and conflict. While recent innovations in stress testing and scenario planning show promise,^30^^,^^47^ these tools are not yet institutionalized across AA.

Lastly, there are promising developments in more flexible and integrated funding approaches, with several pilots underway to develop harmonized MR AA approaches at the country level; however, current funding systems still favor single-hazard approaches, with limited options to adjust actions and to increase funding amounts for MR events. While financing is central to the effectiveness of AA, most funding mechanisms remain formalized and hazard-specific. This rigidity limits the ability to adjust to evolving or compounding risks. Despite growing references to multi-hazard frameworks across agencies, single-hazard practice still dominates. Encouraging differentiated approaches tailored to MR contexts—varying by crisis duration, warning information, and lead time—is critical to promoting cross-agency collaboration in MR AA. Embedding MR frameworks consistently within funding systems will require greater flexibility in trigger methodologies and an institutional acceptance of flexibility in action implementation; yet practitioners also raised concerns that greater flexibility and risk combinations introduce additional complexity. This tension between complexity and flexibility will be key to further exploration to ensure that MR approaches are fit-for-purpose. Nevertheless, experts agree on the importance of moving beyond a single-hazard mindset toward integrated MR management. Some argue for a focus on better connections among hazard-specific frameworks, while others emphasize the potential of impact-focused frameworks.

### Linking MR research with AA practice

Despite advances in MR research,^48^^,^^49^^,^^50^^,^^51^ MR academic analytical frameworks often remain conceptual and disconnected from the operational realities of AA.^14^^,^^48^ Key informant interviews found that these studies often overlook practical constraints, including forecasting limitations, institutional capacity, and decision-making timelines, in data-scarce environments. As a result, analytical frameworks are seldom implemented due to perceived complexity and limited applicability to real-world AA operations.^48^

As highlighted in the previous section, practitioners emphasized the tension between the need for a stronger, systemic, MR lens and the demand for simplicity in the formal approaches employed in AA. Practitioners often question the need to classify MR events based on the various multi-hazard or MR classifications available.^51^ Such classifications are often found to be overly complex, with definitions of categories varying across different fields. Moreover, not all events fit neatly into these classifications or they span multiple categories, further complicating the analysis.

**Figure 2:**
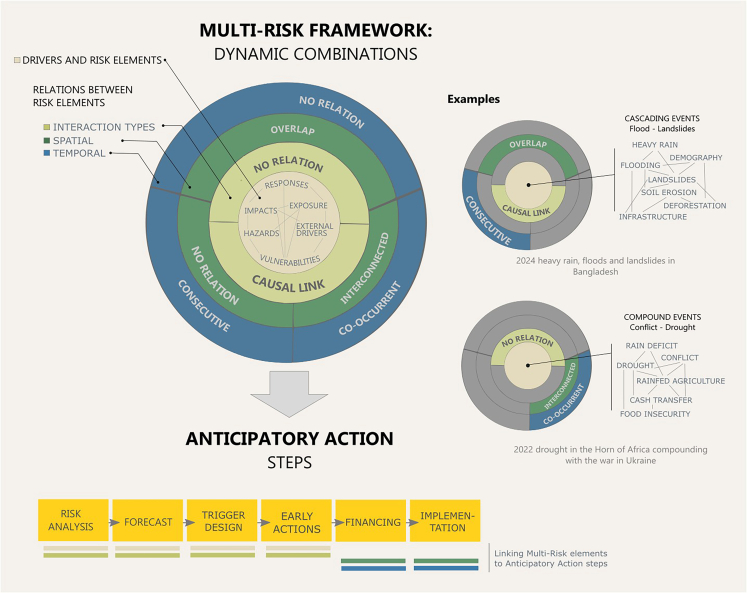
Conceptual diagram illustrating the key characteristics of multi-risk events and their connection to the AA steps The large circle represents the four key characteristics of multi-risk (MR) elements, each shown as a concentric disc: drivers and risk elements (in yellow), interaction types between drivers and risk elements (in light green), spatial relationships (in green), and temporal relationships (in blue). Subcategories within each disc (e.g., no relation, causal link, and overlap) combine to define specific MR events, with examples shown on the right: cascading events (floods and landslides in Bangladesh, 2024) and compound events (conflict and drought in the Horn of Africa, 2022). The bottom part of the figure links each AA step to the relevant MR elements (colored bars) that can support and inform AA planning for that specific step. Source: The authors.

Elucidating key characteristics of MR events for AA practitioners is an essential way to bridge MR research and implementation in AA. Our review of literature on multi-hazard and MR events, alongside interviews and consultation findings, highlights several key characteristics of MR events that are critical for the development of AA frameworks (Figure 2).

• **Diversity in drivers:** Inclusion of a range of diverse risk drivers, including physical and societal drivers leading to impacts (e.g., precipitation, deforestation, disease outbreaks, international and non-international armed conflict).^2^
• **Interaction types:** Considering interaction types within and between risk components (hazard, exposure, and vulnerability), including multiple hazards, as well as interactions between hazards and exposure or vulnerability. These include^4^: (1) independent events occurring without direct influence on each other, (2) causally linked events where one triggers another, or (3) events that alter conditions in a way that increases/decreases the likelihood or severity of subsequent events.
• **Spatial relation:** The spatial distribution of hazards, which may be overlapping, partially overlapping, or separate, include source-to-spread dynamics. It also accounts for spatial dependencies, which shape vulnerability.^52^
• **Temporal relation:** Timing aspects relate to co-occurring events, events occurring shortly after one another, or those separated by large intervals. This also includes the timing of individual slow or rapid onset events.^24^^,^^53^^,^^54^ These temporal relations strongly influence vulnerability, which can compound and cascade across and within sectors,^55^^,^^56^^,^^57^ as well as affect temporal changes in exposure (e.g., displacement).^58^^,^^59^^,^^60^

In practice, any combination of the aforementioned four characteristics (drivers, interaction types, spatial, and temporal relations) shapes the evolution of MR events. These four characteristics are critical to the design of AA frameworks. Information on interaction types between events can inform trigger and threshold identification, as well as the identification of effective early actions. Spatial relations help determine where to implement AA. Temporal relations can inform the time window for mobilizing the financial mechanisms and implementing the related actions.

### Moving forward: Future directions and implications for practice and research

While the understanding of MR dynamics in AA has advanced, the next step is to translate these insights into operational practice through stronger context analysis, next-generation triggers, adaptive financing, and increased investment in readiness for implementation in MR events. Given the current constrained humanitarian funding landscape and the push to expand AA,^13^ it is essential to set clear priorities and ensure that any changes to AA systems are grounded in operational realities. Table 2 and the following subsections outline key actions to bridge science and practice in AA, thereby expanding effective approaches and addressing the challenges identified in cross-cutting challenges in addressing MR across AA section for MR integration in AA.

**Table 2:** Recommendations summary for practitioners, donors, and researchers to strengthen MR integration in AA

Audience	Focus area	Core recommendation
Practitioners	context and risk analysis	map MR typologies to identify relevant hazard-vulnerability combinations; engage communities to understand risk interactions; combine qualitative insights with data-driven tools
	trigger design	pilot flexible, scenario-based triggers capturing compound risks; update vulnerability data through local monitoring; scale actions by impact potential to address non-linear risks
	program design	integrate conflict sensitivity and seasonal scenarios into hazard-specific AA frameworks; link AA with preparedness and recovery phases across the DRM continuum.
Researchers	method development	co-develop MR typologies and dynamic vulnerability indicators with practitioners; prioritize operationally useful models; use storylines, machine learning, and participatory methods to study compound risks
	model validation	test and refine multivariate thresholds (e.g., fire-weather indices and drought-heat models) for anticipatory triggers
Donors/decision-makers	financing instruments	shift from rigid, hazard-specific tools to flexible, forecast-linked financing; align Cat-DDOs and ARC with anticipatory disbursement; fund pilots linking triggers to fiscal relief.
	governance and partnerships	promote joint planning among humanitarian, development, and peace actors; reduce earmarking to enable flexibility; link contingency and resilience finance to support anticipatory and early recovery phases
Crosscutting	capacity and learning	train staff using real MR cases; document and compare pilot evidence to refine models, share lessons, and avoid maladaptation

#### Strengthening MR context analysis

Comprehensive and participatory context analysis is essential for translating MR understanding into AA, as it guides prioritization and adaptation of existing plans. For example, it can identify how single-hazard frameworks should be adapted and where MR integration is most feasible, depending on the likelihood and potential impact of compound events. Analysis of MR contexts can be supported through the framework proposed in Figure 2. Strengthening existing AA frameworks requires first stress-testing them against plausible MR scenarios, enabling stakeholders to recognize connections between events early on to enhance trigger development and early action selection. Not all potential risk combinations or cascading impacts can be formally incorporated into existing frameworks due to challenges such as low probability, perceived complexity, or data gaps. Priority should be given to risk interactions with causal linkages or likely temporal overlap that directly contribute to humanitarian impacts. While some MR scenarios may not always be suitable for immediate framework development, they must be considered in feasibility assessment and preparedness planning, with alternative anticipation strategies explored as part of contingency planning. Combining quantitative and qualitative approaches, including participatory engagement with at-risk communities,^61^^,^^62^ helps assess how different hazards, vulnerabilities, and exposures interact to shape priority risks. Mixed-method tools such as the INFORM risk framework and forensic disaster analysis can support this process, and more methodologies have already been scoped out for AA application.^63^ These approaches have been tested by various humanitarian organizations, but improving spatial granularity and disaggregation still remains critical to ensure local relevance and scalability. We note that while a wide range of MR analysis methods exist,^63^ a key next step for the AA community is to conduct comparative assessments to determine which approaches are most fit-for-purpose, balancing analytical rigor with operational usability.

These operational needs also highlight key research questions for the academic community. The current categorization of MR events requires re-examination to ensure that it effectively supports preparedness and response, as outlined in Section 3. This paper proposes a synthesized research organization framework that focuses on the dimensions of MR dynamics most relevant for AA implementation. Further research should be carried out in close collaboration with the humanitarian community to explore effective strategies for different types of spatial, temporal, and causal relationships between risks. While research on MR events continues to grow, it must evolve to capture the full range of MR dynamics, including rare or unprecedented but high-impact events, as well as the socioeconomic drivers of extreme impacts.^64^ However, this does not imply developing models tailored to narrowly defined or highly specific MR combinations. Instead, approaches should aim for generalizable frameworks that can accommodate diverse risk configurations and enhance scalability. Ultimately, improved operational MR analysis should directly inform trigger design and contingency planning, creating a foundation for MR decision-making.

#### Designing flexible and dynamic trigger systems

Moving from single-hazard to MR AA requires trigger systems that reflect cascading risks and dynamic vulnerability and can be adapted to real-time information. The continued trend of developing and scaling joint trigger frameworks already brings together multiple organizations and hazards. To avoid unnecessary complexity and siloed planning, existing single-hazard plans should be integrated and stress-tested under plausible MR scenarios to ensure interoperability. In Bangladesh, for example, the government-led AA framework for cyclones and cascading flooding brings together various relevant trigger models, and this process has helped centralize coordination and streamline activation decisions.^46^ Operationalizing this vision requires flexible, scenario-based triggers that account for cascading risks and dynamic vulnerabilities. This can build on progress in impact-based forecasting, shifting the focus from individual hazards to impact-level triggers. Triggers for drought or floods should not only consider the hazard itself but also the additional vulnerabilities posed by compounding risks, such as conflict-induced displacement, and potential cascading risks, such as increased likelihood of landslides or wildfires. Developing MHEWSs and multivariate triggers that are both scientifically sound and operationally feasible remains a key priority.^29^ Incorporating real-time indicators, such as socioeconomic conditions, conflict dynamics, and health metrics, can further enhance the relevance and accuracy of triggers. For example, the International Rescue Committee in North-east Nigeria adapted its triggers for flood to support flood-prone farming communities affected by conflict, to ensure that the trigger mechanism design was responsive to the high levels of vulnerability faced by households.^65^ Emerging approaches, such as scenario-based triggers using dynamic or multi-variate thresholds, are being piloted but lack empirical evaluation of their effectiveness. Existing multivariate thresholds include, for example, the fire weather index, a combination of humidity, temperature, precipitation, and wind used in early warning systems (EWSs) for fire risk. This should be closely aligned with the development of MHEWS, given the relatively limited availability of EWSs that account for risk interactions.^29^ Close dialog with forecasting agencies will be crucial to ensure that high-impact, MR dynamics are prioritized in EWS investments under initiatives such as the Early Warnings for All initiative.

Further studies are needed on dynamic vulnerability and methods for quantifying interactions between risk components in data-scarce settings. Approaches such as storylines,^14^ machine learning,^66^ and multivariate statistical analysis,^67^ show promise and should be integrated with community-based, participatory methods to bridge quantitative and qualitative insights.^4^ However, there is a risk of over-fitting or limiting the applicability of models depending on the target populations, making it essential to ensure that these models are adaptable across different contexts and co-created with at-risk communities and first responders. To fully integrate MR triggers, scalability and flexibility must be prioritized. Strengthened localized real-time monitoring systems will be critical to quickly capture and integrate diverse risk signals. Finally, piloting and evaluating integrated trigger systems in real-world MR settings will be essential to refine models, demonstrate feasibility, and strengthen collaboration across AA, public health, and conflict-sensitive programming.

#### Balance trade-offs and ensure flexibility in early actions

While triggers offer a strong entry point for MR integration in AA, this may not be operationally feasible in many contexts due to capacity and data constraints. Instead, adapting operational procedures and being flexible in the types of actions implemented during activations can offer effective pathways to overcome current challenges. For example, in Madagascar, the Red Cross tested their cyclone framework to ensure the procedures would also work under compound flood scenarios, even though a flood AA framework was not yet in place.^30^ Similar adaptive practices were observed during AA activations in the COVID-19 pandemic,^15^ highlighting that flexibility rather than comprehensiveness is often key to readiness. As one expert noted, “To rise to the challenge of MR, we need to think backwards: identify the most critical actions first, then the steps to get there.” This approach underscores the need to prioritize actions that address multiple hazards, prevent cascading impacts, and account for potential trade-offs during activation*.* More research is crucial to explore actions that address trade-offs, avoid maladaptation, and provide low-regret interventions across multiple risk scenarios.^68^ To address the challenges in MR AA in locations where multiple types of risks converge, strategies such as embedding conflict sensitivity principles into hydro-meteorological hazard-focused AA frameworks or developing scenario-based approaches tailored to seasonal calendars can be effective. Furthermore, in many low-resource contexts advance trigger methodologies may be out of scope due to limitations in early warning information or inability to monitor a large number of information sources. In such situations, linking AA with contingency planning is seen as a promising step enabling scenario-thinking for MR preparedness, anticipation, response and recovery. Together, these adaptive strategies shift the emphasis from perfect models to context-appropriate implementation, enabling AA systems to function effectively even amid uncertainty.

#### Address financing barriers by prioritizing flexibility

Financing mechanisms must evolve from hazard-specific instruments toward adaptive systems that can release funds for overlapping or sequential risks. Financing is central to AA, but rigid funding structures and fragmented planning limit adaptability to evolving MR scenarios. Funding for AA varies by organization and scale, ranging from global mechanisms, such as the UN Central Emergency Response Fund (CERF), the Red Cross Disaster Response Emergency Fund (DREF), to localized financing for national actors. Pooled funds and crisis modifiers offer the opportunity to scale up financing in situations where MR events result in severe or extensive impacts, yet this will require adjustments in local legal frameworks to allow institutions to reserve funding for potential scenarios and to activate and disburse higher amounts for multiple risks. Within several organizations that actively implement AA, such as Welthungerhilfe, the Red Cross, the UN, and the START Network, there are discussions on more alignment of existing plans at national scale, which may generate more practical insights into the best strategies. Further analysis is needed to explore the capacity of these funds to support likely MR scenarios and to understand the implications of specific trigger and framework design choices on activation frequency and funding amounts, as this varies significantly across institutions.

Beyond the specific AA funds in the humanitarian sector, two main forms of multi-hazard financing exist: (1) rapid liquidity instruments (e.g., Cat-DDOs and parametric pools) that enable early relief and recovery, and (2) fiscal instruments such as the World Bank’s Climate Resilience Debt clause, which provide short-term debt relief after major events. Aligning these tools with forecast-based approaches can better match the timing and scale of AA funding to multi-hazard realities, where anticipation, response, relief, and recovery for multiple crises might overlap at any given time. Donors such as the Directorate-General for European Civil Protection and Humanitarian Aid Operations (DG ECHO) and the Foreign, Commonwealth and Development Office of the United Kingdom (FCDO) are already promoting MR framing and supporting efforts to integrate single-hazard frameworks into country-level approaches. As more development-focused actors adopt AA principles, new opportunities arise to bridge differing timescales and funding cycles between humanitarian and development programs. This alignment also supports the capacity and tool development needed at national and regional levels to strengthen MHEWS and risk analysis.

As the AA sector moves from pilots to scale, there is an urgent need for more flexible funding models. These should also recognize the different entry points offered by funding actors—for example, bilateral donors for rapid disbursement and diplomacy, multilateral development banks for large-scale contingent finance, and climate funds for concessional resources and resilience standards. Given the ongoing review of the humanitarian system under the Humanitarian Reset,^69^^,^^70^ including the focus on the greatest needs and re-prioritization of countries, a critical review of operational opportunities for AA in complex, MR environments is essential. The growing momentum in climate finance—particularly for hotspots where hydro-meteorological hazards intersect with conflict—offers a promising entry point to overcome current barriers and expand access to AA funding. Ultimately, creating more flexible financing mechanisms that allow rapid, context-specific responses, even in the absence of pre-agreed plans, will be essential to ensure timely AA in complex, MR settings. Prioritization of more connected and integrated planning in AA, recognizing the current challenges as outlined in this paper, will be critical to ensure AA approaches reach impact at scale.

#### Strengthening evidence, learning, and research for MR AA

Embedding continuous learning and evaluation into anticipatory systems is crucial for refining and operationalizing MR approaches. While the expert elicitation and two years of data on AA activations used in this study offer valuable inter-agency insights, they represent only part of the existing experience. Expert participation in this study was skewed toward Africa, Europe, and Asia, with limited representation of the Americas. Expertise of participants in expert elicitation spanned a diverse range of focus areas, including MHEWS, health, conflict-sensitive programming, food security, and displacement. Discussions in global and regional forums on the topic of MR are still dominated by headquarters- and regional-staff and should build more on emerging country-level experiences, such as in Madagascar, Bangladesh, the Philippines, and various countries in Central America. Therefore, a broader dialog is needed to identify effective strategies for integrating MR events into AA operations.

A critical next step is to build a stronger evidence base on the outcomes of incorporating MR dynamics into AA frameworks. Although anecdotal evidence shows that overlooking interlinked risks can lead to missed or ineffective activations, robust empirical evaluation remains limited. This does not imply a lack of value; rather, it highlights an important evidence gap that warrants further research. Furthermore, much of the current learning resides in gray literature and is fragmented, and researchers could add further value by more systematic analyses. Practitioners are, therefore, encouraged to document and share findings from activations, while donors and organizations should actively incentivize these efforts to overcome time and capacity constraints. Such evidence will not only clarify what works in MR settings but also highlight potential trade-offs and design flaws, providing the basis for continuous refinement of AA frameworks in complex and uncertain risk environments. The research organization framework in Figure 2 can help organize this type of research. At the same time, optimizing how existing frameworks are activated, and reviewing cases where multiple activations occur within a single year, can generate practical lessons on coordination and efficiency.^18^^,^^71^ Systematically capturing these experiences can strengthen institutional learning and inform adjustments to operational protocols.

#### Build capacities to collaborate across risk silos

Investing in capacity-building that supports practical, cross-sector learning is essential to operationalize MR AA. Training should use concrete examples to make complex concepts applicable in the field, helping practitioners translate analysis into action. Drawing lessons from broader humanitarian risk analysis and specialized areas, such as conflict sensitivity expertise, can enhance AA frameworks, particularly in fragile, conflict-affected, and vulnerable (FCV) settings where hydro-meteorological hazards are prevalent.

Strengthening capacities must go hand in hand with new forms of collaboration that stretch beyond hazard-silos. Addressing current gaps requires context-specific partnerships beyond the humanitarian sector, engaging local governance structures, traditional leaders, and community-based organizations early in the program design. This ensures that local perspectives are not only heard but are central to decision-making. Cross-sectoral governance and shared learning between DRM, health, agriculture, and peacebuilding actors can further enhance coordination and readiness. At the national level, existing technical working groups and emergency coordination mechanisms offer entry points for improved coordination, and AA framework development offers an opportunity to strengthen exchange and coordination with other actors, as evident from the examples in Bangladesh and Ethiopia, where governments are leading on consolidated multi-agency AA frameworks.

## Conclusions

This paper underscores the need for AA systems that can contend with the realities of MR dynamics—where a wide range of hazards (both hydro-meteorological, environmental, biological, and societal) intersect within different socio-economic contexts. Practitioners and policymakers should embed MR analysis, flexible contingency planning, and dynamic vulnerability monitoring within AA systems, guided by robust context analysis and prioritization of relevant MR scenarios. Four challenges persist: continued emphasis on hydro-meteorological over societal risks, limited methods that balance analytical complexity with usability, difficulties in identifying early actions across cascading events, and financing structures that remain too rigid for evolving risk contexts. Bridging these gaps will require both technical and institutional innovation to design systems that can learn and adapt amid uncertainty.

Practitioners should analyze key hazard-vulnerability patterns and test flexible triggers that scale actions according to their potential efficacy. Researchers can co-develop dynamic vulnerability metrics and validate multivariate thresholds through participatory and data-driven methods. Donors and decision-makers should adopt adaptive, forecast-linked funding that connects contingency and resilience finance. Across all actors, sustained investment in training and shared learning is vital to refine models and avoid maladaptation. Embedding these principles will make AA more adaptive, inclusive, and context-specific, linking preparedness, response, and recovery within systems capable of acting across the complex realities of MR crises. The next frontier is clear: translating MR understanding into anticipatory systems that act early, bridge sectors, and protect people before consecutive and overlapping shocks become disasters.

## Methods

### Study design

This study used a qualitative multi-stage consultation process to examine emerging practices and challenges in integrating multi-risk perspectives into anticipatory action (AA). The research combined three components: (1) a desk review of AA frameworks, (2) semi-structured key informant interviews, and (3) expert elicitation through working group consultations. The process was coordinated through the Anticipation Hub Working Group on Multi-Risk and Anticipatory Action, a multi-stakeholder platform including practitioners, researchers, and donor representatives working on AA and disaster risk management. The objective was to identify trends in AA framework design and synthesise expert perspectives on operational challenges and opportunities for addressing compound and cascading risks

### Desk review of anticipatory action frameworks

A targeted desk review analysed trends in AA framework design and the extent to which frameworks incorporate multi-risk considerations. The review focused primarily on frameworks documented in the Anticipation Hub Global Overview Reports for 2023 and 2024, which compile operational experiences from humanitarian agencies, Red Cross Red Crescent Movement actors, United Nations agencies, and non-governmental organisations.

Two databases of active AA frameworks were analysed: 107 frameworks documented in 2023 and 154 in 2024. For each framework, information was reviewed on hazards addressed, trigger mechanisms, planned anticipatory actions, and references to governance or financing arrangements.

Frameworks were coded to assess hazard coverage, inclusion of multiple hazards as trigger conditions, whether planned actions addressed cascading impacts, and references to institutional or financial arrangements enabling activation. Coding combined deductive categories derived from anticipatory action and multi-hazard disaster risk management literature (e.g., single-hazard and multi-hazard frameworks) with inductive coding to capture emerging operational configurations, including frameworks containing multiple independent hazard plans within a single institutional structure. Coding was conducted by working group members and iteratively reviewed through group discussions to ensure consistency. Findings informed the development of interview guides.

### Key informant interviews

Semi-structured interviews were conducted with 17 experts working on anticipatory action, disaster risk management, multi-risk assessment, and humanitarian financing. Two interview guides were developed: one for practitioners and researchers and one for donor and funding agency representatives.

Participants represented a range of professional roles including humanitarian practitioners, academic researchers, and donor agency representatives. Participants were affiliated with universities, United Nations agencies, non-governmental organisations, donor institutions, and organisations within the International Red Cross and Red Crescent Movement. Participants were based across multiple geographic regions including Europe, Africa, the Americas, and the Middle East.

Interview questions explored experiences with compound and cascading risks, the role of AA in complex risk environments, operational challenges and opportunities for multi-risk approaches, and barriers related to financing and governance. Interviews were conducted remotely between September and December 2024, lasted approximately 45–60 minutes, and were recorded and transcribed. The interview guide is provided in the supplemental information.

This study was conducted as part of a practitioner consultation process to inform a perspective article and did not require formal institutional ethics approval. All participants were informed about the purpose of the study and the intended publication of findings prior to participation. Verbal informed consent was obtained at the start of each interview and written confirmation of consent for the use of anonymized insights in publications was obtained via email following the interview.

Interview data were anonymized, while retaining general descriptors of participants’ professional roles, sector affiliations, and geographic regions.

### Expert elicitation through working group

Additional expert elicitation was conducted through structured discussions within the Anticipation Hub Working Group on Multi-Risk and Anticipatory Action. These consultations were used to validate findings from the desk review, identify emerging operational practices and analytical gaps, and refine interpretations of interview findings.

## Resource availability

### Lead contact

Further information and requests for resources should be directed to the lead contact, Tesse de Boer (2 Institute for Environmental Studies, Vrije Universiteit Amsterdam and Red Cross Red Crescent Climate Centre), boer@climatecentre.org.

### Materials availability

This study did not generate new materials.

### Data and code availability

This study relied on qualitative synthesis of publicly available documentation and expert consultations. The anticipatory action framework databases analysed in this study are compiled in the Anticipation Hub Global Overview Reports for 2023 and 2024, which are publicly available. Interview transcripts are not publicly available due to participant confidentiality.

## Acknowledgments

The working group is grateful to the Anticipation Hub for in-kind support. A.M. has received support from the PerfectSTORM ERC Grant Project (grant no. ERC-2020-StG-948601) and from the VU-UT Alliance, funded through the VU Amsterdam-University of Twente collaboration under the Impact Program Creating Responsible Societies. T.A.d.B. and E.E.C. have received support from the CRAF’d funded project “Multi-hazard Data and Indices for sub-Saharan Africa and MENA” and the Anticipation Hub. C.J.W. was supported by the European Union’s Horizon Europe through the “Multi-hazard and risk informed system for enhanced local and regional disaster risk management (MEDiate)” project under grant agreement no 101074075, and C.J.v.W. was supported by the EU PARATUS project under grant agreement no 101073954. M.C.d.R., R.S.T., and T.T. were supported through the Horizon 2020 MYRIAD-EU Project, funded from the European Union’s Horizon 2020 Research and Innovation Programme (grant no. 101003276).

## Author contributions

Conceptualization, T.A.d.B., A.M., C.J., C.J.v.W., C.J.W., E.P., M.J.C.v.H., M.B., and A.K.; data collection and analysis, T.A.d.B., M.E., and A.M.; writing, T.A.d.B., A.M., M.E., M.J.C.v.H., E.E.C., C.J.W., C.J.v.W., T.T., and A.K.; reviewing and editing drafts, A.K., A.W., B.S., B.L., C.J., C.J.W., C.J.v.W., D.G., E.E.C., K.K., L.J., M.B., M.C.d.R., M.F., M.J.C.v.H., M.V., R.S.T., R.M., T.A., T.A.d.B., and T.T. Aside from the coordination group (T.A.d.B., A.M., E.E.C., and A.K.), all co-authors are ordered alphabetically in the author list.

## Declaration of interests

M.C.d.R. and R.S.T. are guest members of the editorial board of iScience.

## Declaration of generative AI and AI-assisted technologies in the writing process

During the preparation of this work, the authors used ChatGPT to support language editing. After using this tool/service, the authors reviewed and edited the content as needed and take full responsibility for the content of the published article.

## Supplemental information

Document S1. Method S1 and Table S1

## Footnotes

Supplemental information can be found online at https://doi.org/10.1016/j.isci.2026.115618.
