# Supplementary material for: Interdisciplinary perspectives on multi-risk anticipatory action
Source: iScience. 2026 Apr 8;29(7):115618. doi: 10.1016/j.isci.2026.115618 (PMC13320268; doi:10.1016/j.isci.2026.115618)
Supplement: Document S1. Method S1 and Table S1 [file mmc1.pdf]

iScience, Volume *29*

Supplemental information

Interdisciplinary perspectives

on multi-risk anticipatory action

Tesse A. de Boer, Alessia Matanó, Evan Easton-Calabria, Tilly Alcayna, Mirianna Budimir, Marleen C. de Ruiter, Madeline Ewbank, Muhammad Fawwad, Debora Gonzalez, Catalina Jaime, Lindsey Jones, Kim Kristensen, Brenda Lazarus, Rodrigo Mena, Edward Parkinson, Robert Šakić Trogrlić, Beth Simons, Timothy Tiggeloven, Marc J.C. van den Homberg, Cees J. van Westen, Martha M. Vogel, Andy Wheatley, Christopher J. White, and Andrew Kruczkiewicz

Methods S1. Supplementary materials STAR methods

Set of questions for semi-structured interviews with practitioners and researchers:

1. What is your role within your organisation?
2. Please tell us about your experience with compound and cascading risk/crises in disaster risk management.
3. According to your expertise, where do you foresee AA's role in compounding and cascading crises?
4. What are the most pressing gaps and challenges for implementing AA in the context of compounding and cascading crises?
5. Which opportunities do you anticipate for implementing anticipatory action for compound crises?
6. What would be most helpful in implementing anticipatory action for compound crises better? Can you share experiences where anticipatory action for compound and/or cascading crises has been implemented at the operational level?
7. Is there additional research that should take place? (*And if so, what are the specific needs and barriers?*)
8. What are (or could be) challenges with funding and donors for AA and compound/cascading crises?

Set of questions for semi-structured interviews with donor and funding agency personnel:

1. What is your role within your organisation?
2. Could you tell me more about your experience with financing and supporting anticipatory action programming?
3. What is your agency's current approach to funding anticipatory action projects in complex, multi-hazard environments?
4. Do you have any examples of projects where your agency supported an AA project that addressed multiple risks? What made these cases possible?
5. How do funding mechanisms encourage or limit flexibility in anticipatory action? (*Do you have any examples of this?)*
6. What are the most pressing gaps and challenges for improving AA funding structures in settings with compounding and cascading risks?
7. Where do you see opportunities for progress?
8. Is there additional research that should take place? (And if so, what are the specific needs and barriers?)

Table S1. Participants to the semi-structured interviews

No.	Designation	Type of Organization	Office Region	Date of Interview
1	Practitioner	International Red Cross Red Crescent Movement	Europe	13/09/2024
2	Researcher	University	Europe	16/09/2024
3	Practitioner	International Red Cross Red Crescent Movement	Europe	17/09/2024
4	Researcher	University	Europe	17/09/2024
5	Practitioner	International Red Cross Red Crescent Movement	North America	18/09/2024
6	Practitioner	International Red Cross Red Crescent Movement	South America	18/09/2024
7	Practitioner	International Red Cross Red Crescent Movement	West Africa	19/09/2024
8	Practitioner	UN Agency	East Africa	20/09/2024
9	Practitioner	UN Agency	East Africa	
10	Practitioner, researcher	INGO, Think tank	Europe	23/09/2024
11	Practitioner	International Red Cross Red Crescent Movement	Middle East	7/10/2024
12	Practitioner	International Red Cross Red Crescent Movement	Americas	9/10/2024
13	Researcher	International Red Cross Red Crescent Movement	Europe	9/10/2024
14	Donor agency personnel	UK Agency	Europe	10/12/2024

15	Donor agency personnel	EU Agency	East Africa	11/12/2024
16	Pracititioner	NGO	East Africa	15/12/2024
17	Researcher	University	Americas	16/12/2024
